# Capsaicin enhances chemosensitivity and mitigates MMP11-driven aggressiveness in human bladder cancer

**DOI:** 10.1186/s12935-026-04397-x

**Published:** 2026-07-24

**Authors:** Te-Fu Tsai, Hao-Chiun Chang, Yen-You Lin, Yen-Chen Chen, Win-Ping Deng, Thomas I-Sheng Hwang, Kuang-Yu Chou, Chao-Yen Ho, Hung-En Chen, Chih-Hsin Tang, Shih-Wei Wang, An-Chen Chang

**Affiliations:** 1 Division of Urology, Department of Surgery, Shin Kong Wu Ho-Su Memorial Hospital, Taipei, 111045 Taiwan, ROC; 2 Division of Urology, School of Medicine, Fu-Jen Catholic University, New Taipei, 242062 Taiwan, ROC; 3 Department of Medicine, MacKay Medical University, New Taipei, 252005 Taiwan, ROC; 4 Division of Sports Medicine & Surgery, Department of Orthopedic Surgery, MacKay Memorial Hospital, 104217 Taipei, Taiwan, ROC; 5 Translational Medicine Center, Research Department, Shin Kong Wu Ho-Su Memorial Hospital, Taipei, 111045 Taiwan, ROC; 6 Institute of Translational Medicine and New Drug Development, China Medical University, Taichung, 406040 Taiwan, ROC; 7 Graduate Institute of Medical Sciences, College of Medicine, Taipei Medical University, Taipei, 110301 Taiwan, ROC; 8 School of Dentistry, College of Oral Medicine, Taipei Medical University, Taipei, 110301 Taiwan, ROC; 9 Stem Cell Research Center, College of Oral Medicine, Taipei Medical University, Taipei, 110301 Taiwan, ROC; 10 Department of Urology, Taipei Medical University, Taipei, 110301 Taiwan, ROC; 11 Institute of Traditional Medicine, School of Medicine, National Yang-Ming University, Taipei, 112304 Taiwan, ROC; 12 Department of Pharmacology, School of Medicine, China Medical University, Taichung, 406040 Taiwan, ROC; 13 Chinese Medicine Research Center, China Medical University, Taichung, 406040 Taiwan, ROC; 14 Department of Medical Laboratory Science and Biotechnology, College of Medical and Health Science, Asia University, Taichung, 413305 Taiwan, ROC; 15 Institute of Biomedical Sciences, Mackay Medical University, New Taipei, 252005 Taiwan, ROC; 16 School of Pharmacy, Kaohsiung Medical University, Kaohsiung, 807378 Taiwan, ROC; 17 School of Oral Hygiene, College of Oral Medicine, Taipei Medical University, No.250, Wuxing St., Xinyi Dist, Taipei, 110301 Taiwan, ROC

**Keywords:** Bladder cancer, Capsaicin, MMP11, Chemosensitivity, Invasiveness

## Abstract

Bladder cancer (BLCA) is the second most common malignant cancer of the male urological system. BLCA is characterized by a high rate of recurrence. Capsaicin is increasingly being used in cancer treatment. This study analyzed the inhibitory effects of capsaicin on cell invasiveness and examined whether capsaicin enhances the chemosensitivity of BLCA. High concentrations of capsaicin induced apoptosis in cancer cells. Co-administration of capsaicin with various chemotherapy drugs (cisplatin, epirubicin, and mitomycin C) increased cancer cell sensitivity to chemotherapy. At lower concentrations, capsaicin effectively inhibited cell invasiveness by downregulating MMP11 expression. Mechanistically, capsaicin suppressed MMP11 expression through the transient receptor potential vanilloid 1 receptor and the p38/extracellular signal-regulated kinase/c-Jun signaling pathway. Functional analysis revealed a positive correlation between the expression level of MMP11 and various ontogenetic properties of BLCA. MMP11 expression was positively correlated with clinical staging, muscle-invasive bladder cancer, lymphatic metastasis, and poor prognosis in patients with BLCA. These findings suggest that MMP11 has potential as a clinical prognostic marker for BLCA. Finally, capsaicin demonstrated significant tumor growth suppression and enhanced chemosensitivity in vivo. Taken together, capsaicin may promote apoptosis, inhibit cell invasion in vitro, and suppress tumor growth in vivo. Combining capsaicin with first-line chemotherapeutic drugs may enhance their efficacy against BLCA, and therefore, which indicates that capsaicin has promise as a clinical medication.

**Supplementary Information:**

The online version contains supplementary material available at https://doi.org/10.1186/s12935-026-04397-x.

## Background

According to clinical statistics, bladder cancer (BLCA) is the second most prevalent type of malignant cancer of the male urological system, following prostate cancer [1]. Unlike other types of cancers, BLCA has a high rate of recurrence [2]. Statistical evidence indicates that non–muscle invasive bladder cancer (NMIBC) has a 5-year recurrence rate of approximately 65% and a survival rate of 95% [3]. Muscle-invasive (MIBC) and metastatic bladder cancers are associated with considerably lower survival rates (approximately 50% and 5%, respectively) [4, 5]. However, a comprehensive investigation of the mechanisms underlying BLCA progression may facilitate the development of novel therapeutic strategies, which could ultimately improve clinical treatments and the prognosis for BLCA.

Degradation of the constituents of the extracellular matrix is caused by a collection of zinc-dependent endopeptidases—matrix metalloproteinases (MMPs) [6]. The MMP family has been implicated in various physiological and pathological phenomena, including angiogenesis [7], tissue remodeling [8], wound healing [9], tumor growth and metastasis [10–12]. MMP11 (stromelysin-3), a component of the MMP family, is typically expressed at low levels in normal cells but shows high expression levels in various cancer cells, including breast cancer [13], lung adenocarcinoma [14], and colon cancer [15]. In vitro and in vivo studies have revealed that the inhibition of MMP11 expression can impede the growth of gastric tumors [16] and prevent the metastasis of hepatocarcinoma to lymph nodes [17]. Overexpression of MMP11 has also been observed among invasive primary carcinomas and metastases associated with breast cancer [18]. Furthermore, studies have indicated that the level of MMP11 expression may serve as an indicator for patients at high risks of recurrent cancers, such as pancreatic cancer, colon cancer, and breast cancer [19, 20]. A correlation between the occurrence of MMP11 rs131451 polymorphic variants and that of advanced-stage tumors as well as high-risk D’Amico classification was identified in patients with prostate cancer who had biochemical recurrence [21]. Given the potential significance of MMP11 in various cancers, further studies must be conducted to enable understanding of MMP11 as a viable target for cancer treatment and development of targeted therapies that are effective and safe.

Capsaicin is a derivative of homovanillic acid and the key pungent compound of chili peppers [22]. Capsaicin has gained worldwide recognition for its culinary use as a spice and medicinal use as an analgesic [23, 24], antioxidant [25], anti-inflammatory [26], and anti-obesity agent [27]. In 2021, David Julius was awarded the Nobel Prize in Physiology or Medicine for his discovery of the capsaicin receptor protein that activates the transient receptor potential vanilloid 1 (TRPV1) cation channel and thereby causes heat and pain sensations on the skin [28]. The identification of TRPV1 not only clarified the mechanisms underlying pain but also facilitated the development of therapeutic interventions against inflammatory conditions and for the alleviation of symptoms such as pain [29]. Capsaicin has been extensively studied for its anticancer properties, including its ability to induce apoptosis, inhibit cell growth, and prevent angiogenesis and metastasis [30]. In addition, capsaicin exhibits both chemopreventive and chemotherapeutic properties [31, 32]. It has been used to manage postsurgical neuropathic pain and oral mucositis in patients with cancer [33, 34]. However, the understanding of how capsaicin interacts with the TRPV1 receptor in human cancers remains incomplete.

In the current study, we investigated the potential benefits of capsaicin in terms of the inhibition of BLCA’s hallmark features, such as proliferation, apoptosis, and invasion in vitro and tumor growth in vivo. We further explored whether combining capsaicin with first-line chemotherapeutic drugs enhances their efficacy against BLCA.

## Methods

### Cell culture and siRNA transfection

The human BLCA cell lines UMUC3 (RRID: CVCL_1783) and T24 (RRID: CVCL_0554) were acquired from the Bioresource Collection and Research Center (Hsinchu, Taiwan) and cultured in Minimal Essential Medium (cat. no. 12492013; Gibco, Thermo Fisher Scientific, Inc.) and McCoy’s 5 A medium (cat. no. 16600082; Gibco, Thermo Fisher Scientific, Inc.), respectively; both media were supplemented with 10% fetal bovine serum (cat. no. A5670701; Gibco, Thermo Fisher Scientific, Inc.), 2 mM GlutaMAX-1 (cat. no. 35050061; Gibco, Thermo Fisher Scientific, Inc.), 100 U/mL penicillin, 100 µg/mL streptomycin and 0.25 µg/mL amphotericin B solution (cat. no. 516104-M; Sigma-Aldrich/Merck KGaA). The cells were incubated at 37 °C in a humidified atmosphere under 5% CO_2_.

A pool of ON-TARGETplus control nontargeting siRNA and human MMP11 siRNA (Dharmacon) was used for transfection. To transfect 10 nM of each siRNA into the BLCA cells, the ViaFect transfection reagent (cat. no. E4981; Promega) was used as per the manufacturer’s instructions.

### Colony formation

The BLCA cells were seeded onto a six-well plate (density, 3 × 10^3^ cells/well). The cells were then treated with different concentrations of capsaicin or chemotherapeutic drugs for 1 week. After the fixation process with 3.7% formaldehyde (cat. no. 252549; Sigma-Aldrich/Merck KGaA) for 20 min, the colonies were further stained with 0.5% crystal violet (w/v) for an additional 20 min. The number of colonies was quantified by measuring the absorbance of crystal violet extracted with 10% acetic acid.

### Western blot analysis

The immunoblotting method was identical to that used in our previous study [35]. Primary anti-cleaved caspase-3 (cat. no. GTX636810; GeneTex), anti-cleaved poly (ADP-ribosyl) transferase (PARP; cat. no. GTX636804; GeneTex), anti-MMP11 (cat. no. GTX05076; GeneTex), anti-CDH11 (cat. no. GTX04544; GeneTex), anti-MMP2 (cat. no. GTX104577; GeneTex), anti-vimentin (cat. no. ab92547; Abcam), anti-twist (cat. no. ab50887; Abcam), or anti-GAPDH (cat. no. GTX100118; GeneTex) were used to detect protein levels.

### Evaluation of cell motility and invasive capacity

For gap closure assay, a three-well silicone insert was obtained from ibidi (cat. no. 80366) and used to create wound-like scratches in a 12-well plate. BLCA cells were incubated with different concentrations of capsaicin for 24 h. Images of migrated cells were captured using a microscope (Zeiss), and the distance between the edges of each scratch was measured using ImageJ.

Furthermore, Transwell migration and invasion assay were evaluated by using Transwell inserts (pore size, 8 μm; Costar) placed in 24-well plates and by following the methodology described in our previous study [36]. Images of the migrated and invaded cells were captured under 200× magnification by using the Eclipse Ti2 microscope (Nikon).

### Flow cytometric detection of apoptosis

The BLCA cells were incubated with capsaicin for 24 h. Apoptotic cells were assessed using a fluorescein isothiocyanate annexin V apoptosis detection kit (BD Biosciences) in accordance with the manufacturer’s instructions. Cell populations were categorized as live, early apoptotic, and late apoptotic by using our previously reported method [37] and were quantified using the Accuri C5 flow cytometer (BD Biosciences).

### Bioinformatic analysis

The Cancer Genome Atlas Urothelial Bladder Carcinoma (TCGA-BLCA) dataset was analyzed to enable determination of the clinical significance of MMP11 in various clinical stages, T status, and lymphatic metastasis. The Gene Expression Profiling Interactive Analysis 2 (GEPIA2) web server was used to evaluate overall survival (OS) and disease-free survival (DFS) in patients with BLCA based on high or low MMP11 expression levels. The cBioportal platform was used to investigate the correlations between the expression levels of MMP11, TRPV1, mitogen-activated protein kinase (MAPK)11, MAPK1, and JUN in human BLCA tissues. The Cancer Cell Line Encyclopedia (CCLE) database was analyzed to evaluate TRPV family expression in the BLCA cells. Analysis of the Gene Expression Omnibus (GEO) database (GDS1479/203878_s_at) was used to determine MMP11 expression levels in superficial transitional cell carcinoma and MIBC. MMP11 expression in different T statuses (T1–T4) was further analyzed using GEO dataset (GDS4456/203878_s_at). For human BLCA, functional analysis of MMP11 was conducted using the WEB-based GEne SeT AnaLysis Toolkit.

### Subcutaneous tumor model

The animal study protocols were approved by the Institutional Animal Care and Use Committee (No. 110SKH005); the animal experiments were performed in compliance with the Guidelines of Animal Experimentation set forth by Shin Kong Wu Ho-Su Memorial Hospital. The animal model involved 6-week-old male nu/nu mice. In brief, UMUC3 cells (1 × 10^6^) were combined with a mixture of complete medium and Matrigel (1:1) and subcutaneously introduced into the mice. After 1 week of bladder tumor stabilization, the mice were treated with cisplatin (2 mg/kg), capsaicin (2.5 mg/kg), or a combination of cisplatin (2 mg/kg) and capsaicin (1 mg/kg). The dosages used for capsaicin and cisplatin were determined based on previous studies [38, 39]. The tumor size was manually measured every week. After 3 weeks of treatment, the tumor tissues were collected to enable evaluation of the expression levels of the indicated proteins through immunohistochemical staining.

### Immunohistochemistry

A human BLCA tissue array (BL2081a) comprising 208 samples was acquired from US Biomax. The Novolink Polymer Detection System (cat. no. RE7140-CE; Leica Biosystems) was used to stain the human tissue array and mouse tumor tissues with anti-MMP11 (Cell Signaling) as per the manufacturer’s instructions. In addition, mouse tumor tissues were stained with various primary antibodies, including anti-MMP11 (cat. no. GTX05076; GeneTex), anti-CDH11 (cat. no. GTX04544; GeneTex), anti-MMP2 (cat. no. GTX104577; GeneTex), anti-vimentin (cat. no. ab92547; Abcam), anti-twist (cat. no. ab50887; Abcam), and anti-VEGFC (cat. no. GTX32962; GeneTex). The staining results were evaluated using ImageJ; three pathologists independently evaluated the percentage of tumor tissue by using positive staining (ranging from 0 to 100%) and determined the staining intensity (0, negative; 1, weak; 2, moderate; 3, strong). These assessments were combined to generate a final staining score, which ranged from 0 to 300.

### Statistical analysis

All experiments were conducted in triplicate. The Student’s *t*-test was performed to compare two groups, and one-way or two-way analysis of variance (ANOVA) with Tukey’s multiple comparison test was used to compare three or more groups. Correlations were analyzed using Spearman’s test and are presented as *P* values and correlation coefficients (r). Overall survival was analyzed using Kaplan–Meier survival analysis, and group differences were evaluated using the log-rank test, with hazard ratios estimated by the Cox proportional hazards model. The data are presented in terms of mean ± standard deviation values. Statistical significance was set at *P* < 0.05.

## Results

### Capsaicin exerts anti-tumor effects through induction of apoptosis and inhibition of tumor growth

**Fig. 1:**
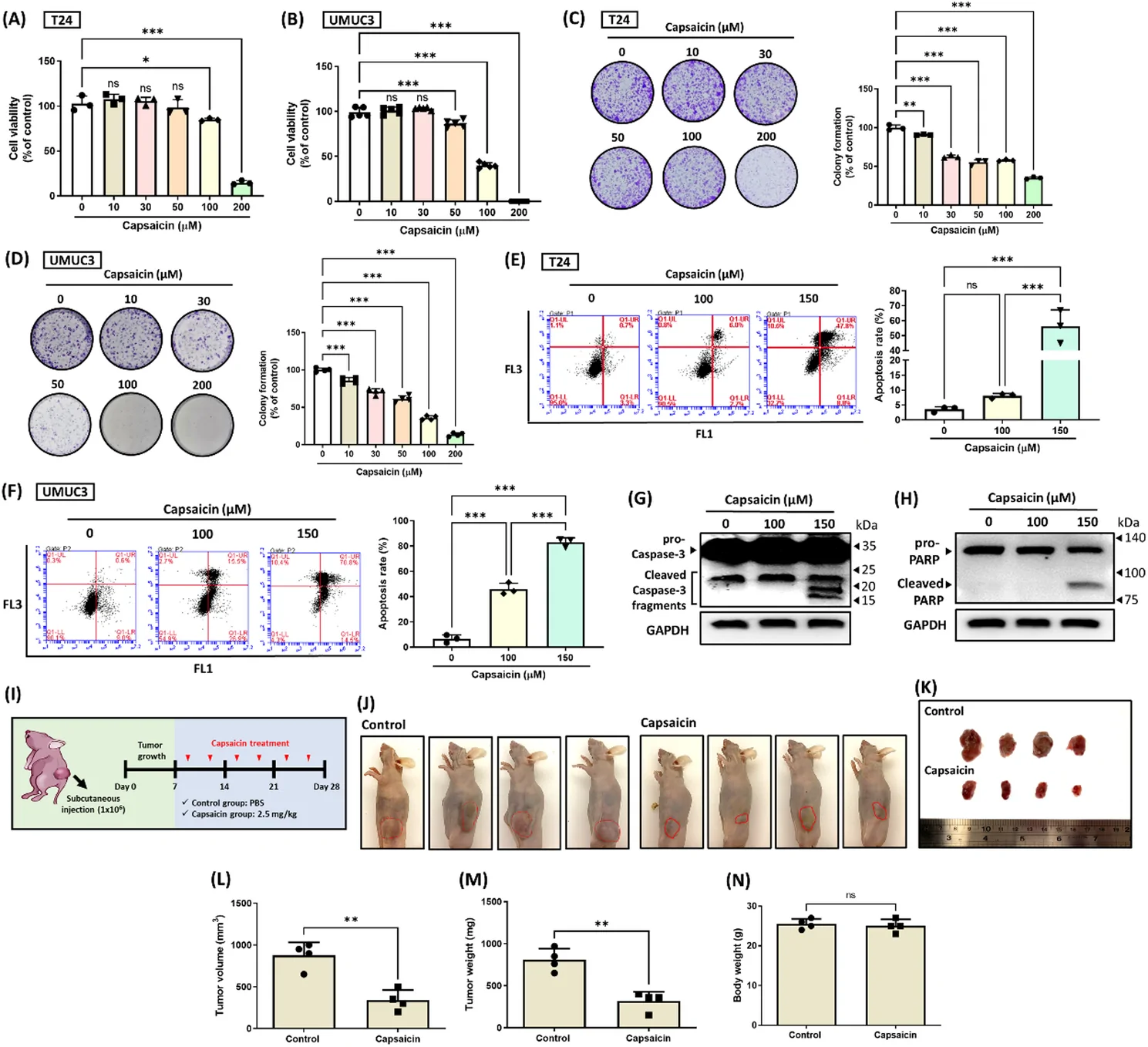
Capsaicin promotes in vitro apoptosis and in vivo tumor suppression. (**A&B**) BLCA cells were treated with different concentrations of capsaicin for 24 h to assess cell viability. (**C&D**) The survival rate of BLCA cells treated with capsaicin for 1 week was evaluated through colony formation assay. (**E&F**) The effects of capsaicin on apoptosis were investigated using a fluorescein isothiocyanate annexin V apoptosis detection kit. (**G&H**) The expression levels of cleaved caspase-3 (15/17 kDa) and cleaved PARP (89 kDa) were evaluated through Western blot analysis. (**I**) Flowchart of the establishment of the in vivo tumor-xenograft mouse model with control and capsaicin groups (4 mice per group). (**J**) Images of tumor-bearing mice treated with capsaicin or left untreated. (**K**) Representative images of tumors excised from each group. (**L–N**) Tumor volume, tumor weight, and mouse body weight were recorded manually and quantified. NS, not significant; * *P* < 0.05, ** *P* < 0.01, and *** *P* < 0.001 compared with the control group, as determined by one-way ANOVA (**A–F**) or Student’s *t*-test (**L–N**)

We first evaluated the effects of various concentrations of capsaicin on two human BLCA cell lines (T24 and UMUC3) to determine cell viability. High concentrations of capsaicin (100 and 200 µM) significantly reduced the viability of BLCA cells, whereas low concentrations exerted weak effects (Fig. 1A&B). The colony formation assay revealed that capsaicin reduced the survival rate of BLCA cells after week-long incubation (Fig. 1C&D). The T24 cells treated with 200 µM capsaicin exhibited a 50% reduction in survival (Fig. 1C); similar results were obtained for the UMUC3 cells treated with 100 µM capsaicin (Fig. 1D). Furthermore, capsaicin induced apoptosis in the BLCA cells in a dose-dependent manner (Fig. 1E&F). The activation of caspase-3, a crucial protein in the apoptotic pathway [40], and the subsequent cleavage of PARP, one of the downstream targets of caspase-3 [41], are key signaling events that play pivotal roles in initiating apoptosis. Our results demonstrated that capsaicin decreased the expression of the anti-apoptotic protein BCL2 without significantly affecting BAX expression (Supplementary Fig. 1A and 1B). The BAX/BCL2 ratio was markedly increased following capsaicin treatment (Supplementary Fig. 1C). Moreover, capsaicin increased the levels of cleaved caspase-3 and cleaved PARP protein expression (Fig. 1G and H), indicating the activation of apoptotic signaling and induction of apoptosis in BLCA cells. These results indicate that high concentrations of capsaicin induce apoptosis in BLCA cells, consequently reducing the survival rate.

The inhibitory effects of capsaicin on the growth of BLCA tumors were validated using an in vivo animal model. BLCA cells were subcutaneously injected into the study mice, which were then treated intraperitoneally with capsaicin twice a week (Fig. 1I). Capsaicin effectively suppressed the growth of BLCA tumors (Fig. 1J&K), leading to significant reductions in tumor volume and weight (Fig. 1L&M). However, capsaicin did not affect the overall body weight of the mice (Fig. 1N). Collectively, these findings indicate that capsaicin suppresses BLCA tumor growth in vivo.

### Capsaicin enhances the efficacy of chemotherapeutic drugs against BLCA

**Fig. 2:**
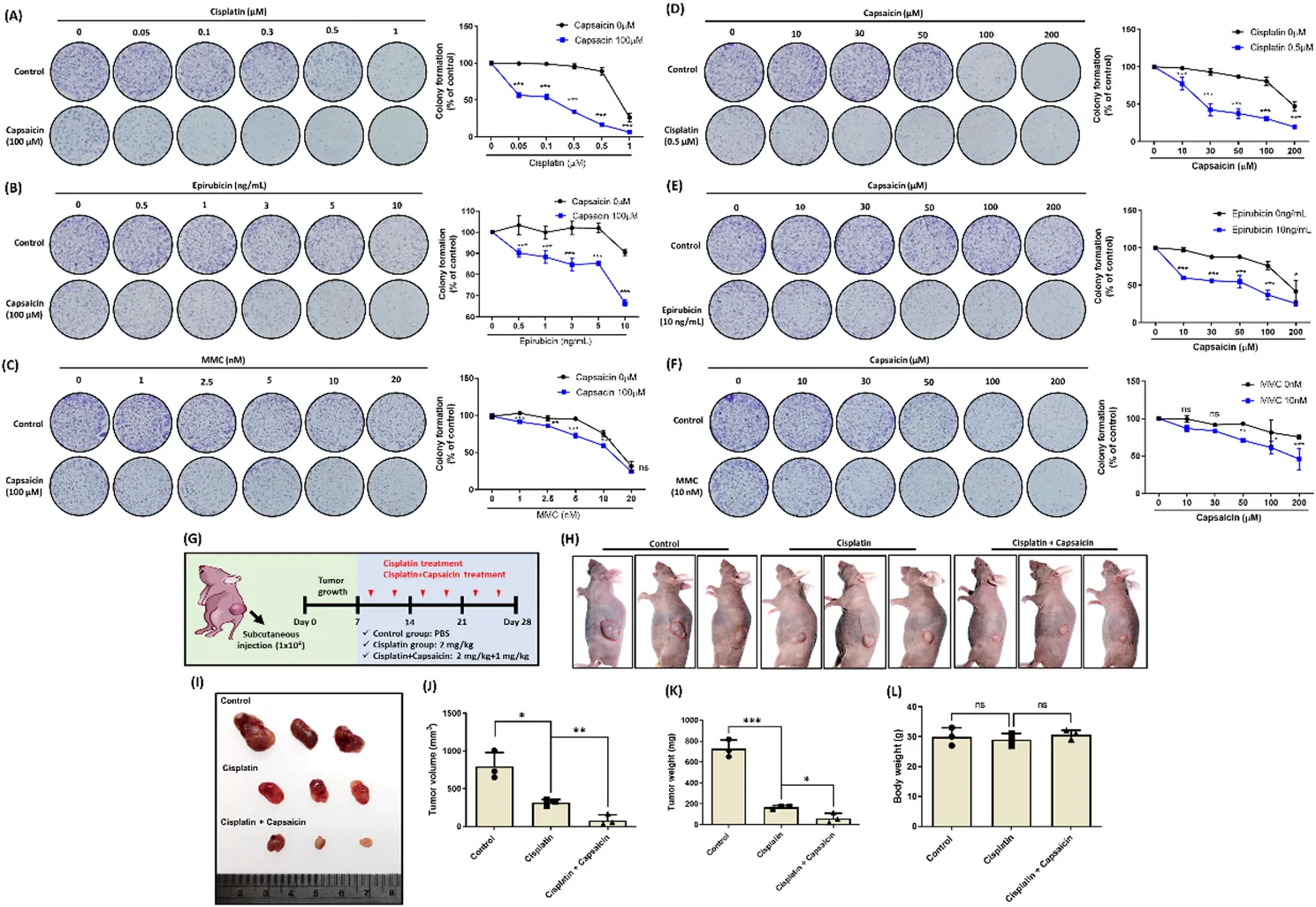
Co-administration of cisplatin and capsaicin improves chemotherapy treatment for BLCA. (**A–C**) Colony formation assay was performed to evaluate the survival rate of BLCA cells treated with various concentrations of chemotherapeutic drugs (cisplatin, epirubicin, mitomycin c) with or without capsaicin (100 µM) for 1 week. (**D–F**) Coadministration of BLCA cells with chemotherapeutic drugs and varying doses of capsaicin (0 ~ 200 µM). A colony formation assay was performed after 1 week. (**G**) Flowchart of the establishment of the in vivo tumor-xenograft mouse model (3 mice per group). (**H**) Images of tumor-bearing mice treated with cisplatin alone or in combination with capsaicin. (**I**) Representative images of tumors excised from each group. (**J–L**) Mouse body weight, tumor volume, and tumor weight were recorded manually and quantified. NS, not significant; * *P* < 0.05, ** *P* < 0.01, and *** *P* < 0.001 compared with the control group, as determined by two-way ANOVA (**A-F**) or one-way ANOVA (**J-L**)

The side effects of chemotherapeutic drugs are a major challenge in clinical practice [42]. We investigated whether capsaicin enhances the sensitivity of BLCA cells to several chemotherapeutic drugs, including cisplatin, epirubicin, and mitomycin c (MMC). Firstly, we combined different doses of chemotherapeutic drugs with capsaicin; unlike treatment with chemotherapeutic drugs alone, combination treatment significantly reduced the survival rate of the cancer cells (Fig. 2A-C). Next, the chemotherapeutic drugs were combined with varying concentrations of capsaicin. As the concentration of capsaicin increased, the chemotherapeutic drugs demonstrated heightened sensitivity, indicating a positive correlation between capsaicin levels and drug effectiveness (Fig. 2D-F). Moreover, the combination treatment of capsaicin with chemotherapeutic drugs had a synergistic effect (CI < 1) (Supplementary Table 1).

To evaluate the effect of cisplatin alone and in combination with capsaicin on tumor growth, an in vivo animal model was used (Fig. 2G). After 3 weeks of treatment, tumor mass was assessed at the end of the study (Figs. 2H&I). Mice treated with cisplatin alone showed a marked reduction in tumor volume; however, those treated with both cisplatin and capsaicin exhibited the most substantial tumor shrinkage, with tumors visibly smaller than those in the other groups, indicating that capsaicin effectively boosted the anti-tumor efficacy of cisplatin (Fig. 2J-L). Taken together, these findings indicate that capsaicin has potential as an adjunctive therapeutic agent for enhancing the efficacy of current first-line chemotherapeutic drugs against BLCA.

### Capsaicin reduces the aggressiveness of cancer cells by downregulating MMP11 expression

**Fig. 3:**
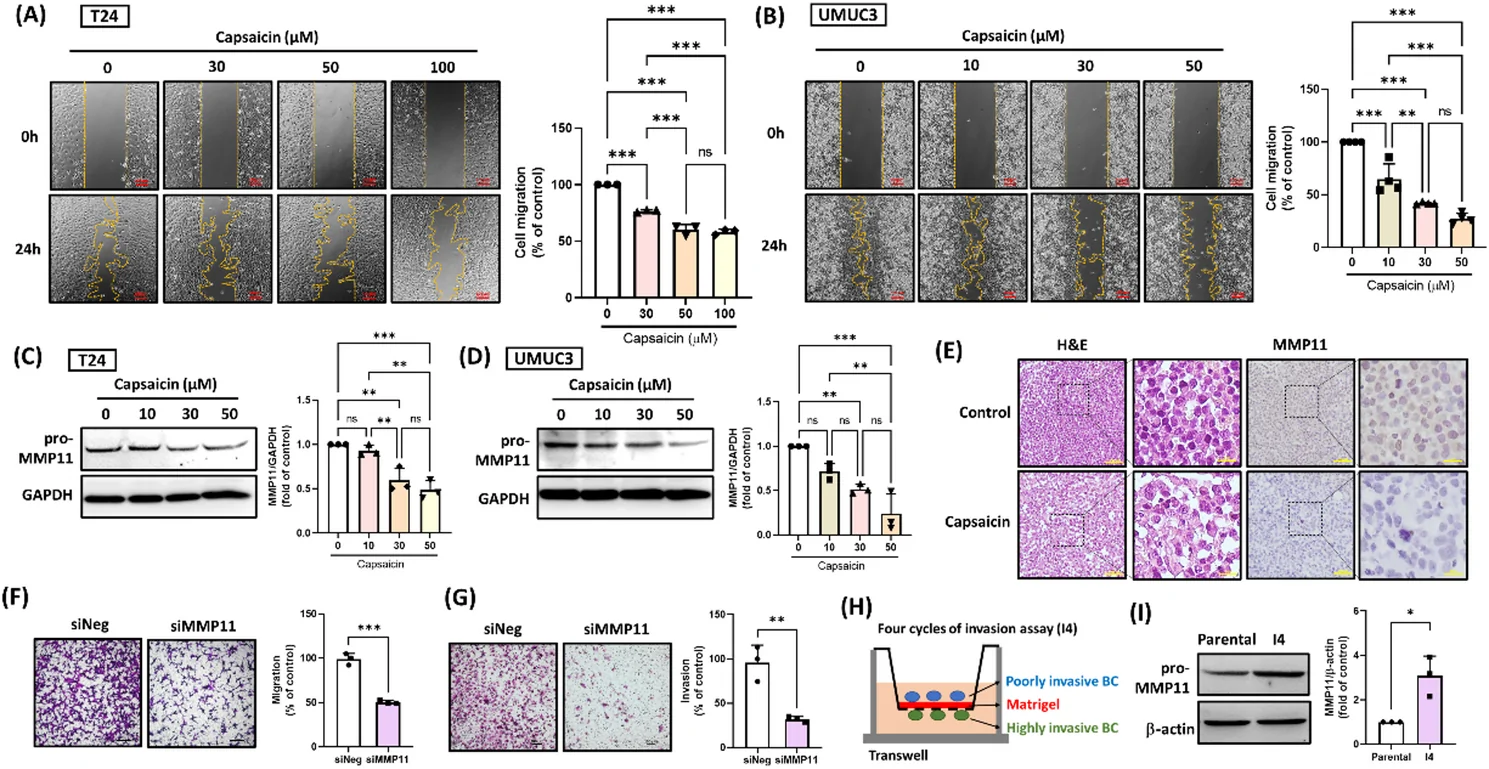
Capsaicin suppresses cancer cell aggressiveness by targeting MMP11. (**A&B**) BLCA cells were treated with varying concentrations of capsaicin for 24 h; the effect of capsaicin on cell motility was assessed through gap closure assay. Scale bar = 100 μm. (**C&D**) MMP11 levels were evaluated through Western blot analysis. (**E**) Histologic sections of the tumors were stained with H&E and MMP11 in the mouse tumor tissues. Scale bar = 25, 100 μm. (**F&G**) BLCA cells were transfected with either negative control siRNA (siNeg; 10 nM) or MMP11 siRNA (siMMP11; 10 nM). Cell motility and invasiveness were analyzed through Transwell migration and invasion assay, respectively. Scale bar = 100 μm. (**H**) Procedure for establishing a highly invasive BLCA cell line (referred to as I4) through Transwell invasion assay. (I) MMP11 levels were evaluated through Western blot analysis for both the parental and I4 cells. NS, not significant; * *P* < 0.05, ** *P* < 0.01, and *** *P* < 0.001 compared with the control group, as determined by one-way ANOVA (**A–D**) or Student’s *t*-test (**F, G, I**)

The migration and invasiveness of cancer cells are closely associated with distant metastasis [43]. Therefore, we investigated whether capsaicin reduces the motility of BLCA cells as a means of assessing its therapeutic potential against distal metastasis. The gap closure assay results revealed that capsaicin inhibited the migration of BLCA cells in a concentration-dependent manner (Fig. 3A&B). To further explore potential metastasis-related genes associated with BLCA progression, we analyzed gene expression profiles using the UALCAN web platform based on the TCGA-BLCA dataset. Among all analyzed genes, MMP11 showed the most significant upregulation in tumor tissues compared with normal tissues, ranking first among the significantly overexpressed genes (Supplementary Fig. 2A). Previous studies have demonstrated that MMP11 plays a critical role in regulating cancer cell proliferation, migration, invasion, and metastasis across various cancer types [44, 45]. However, whether capsaicin suppresses the invasive potential of bladder cancer cells through negative regulation of MMP11 remains unclear. In the present study, we discovered that low concentrations of capsaicin (30–50 µM) downregulated the protein levels of MMP11 expression (Fig. 3C&D). Importantly, immunohistochemical staining showed that capsaicin reduced MMP11 expression levels in mouse tumor tissues (Fig. 3E).

To clarify the role of MMP11 in regulating the motility and invasiveness of BLCA cells, we performed siRNA-mediated knockdown of endogenous MMP11. This knockdown significantly reduced both migration and invasion of T24 cells, indicating that MMP11 plays a role in enhancing BLCA aggressiveness (Fig. 3F&G). Furthermore, we established a highly invasive BLCA cell line (I4) through four sequential rounds of Transwell invasion selection (Fig. 3H). In I4 cells, MMP11 expression was markedly upregulated at protein levels, supporting a positive correlation between MMP11 expression and invasive potential (Fig. 3I). Taken together, the results indicate that capsaicin-mediated inhibition of MMP11 contributes to reduced migration and invasiveness of the BLCA cells.

### TRPV1 receptor and MAPK signaling molecules are involve in capsaicin-induced reduction of BLCA cell aggressiveness

**Fig. 4:**
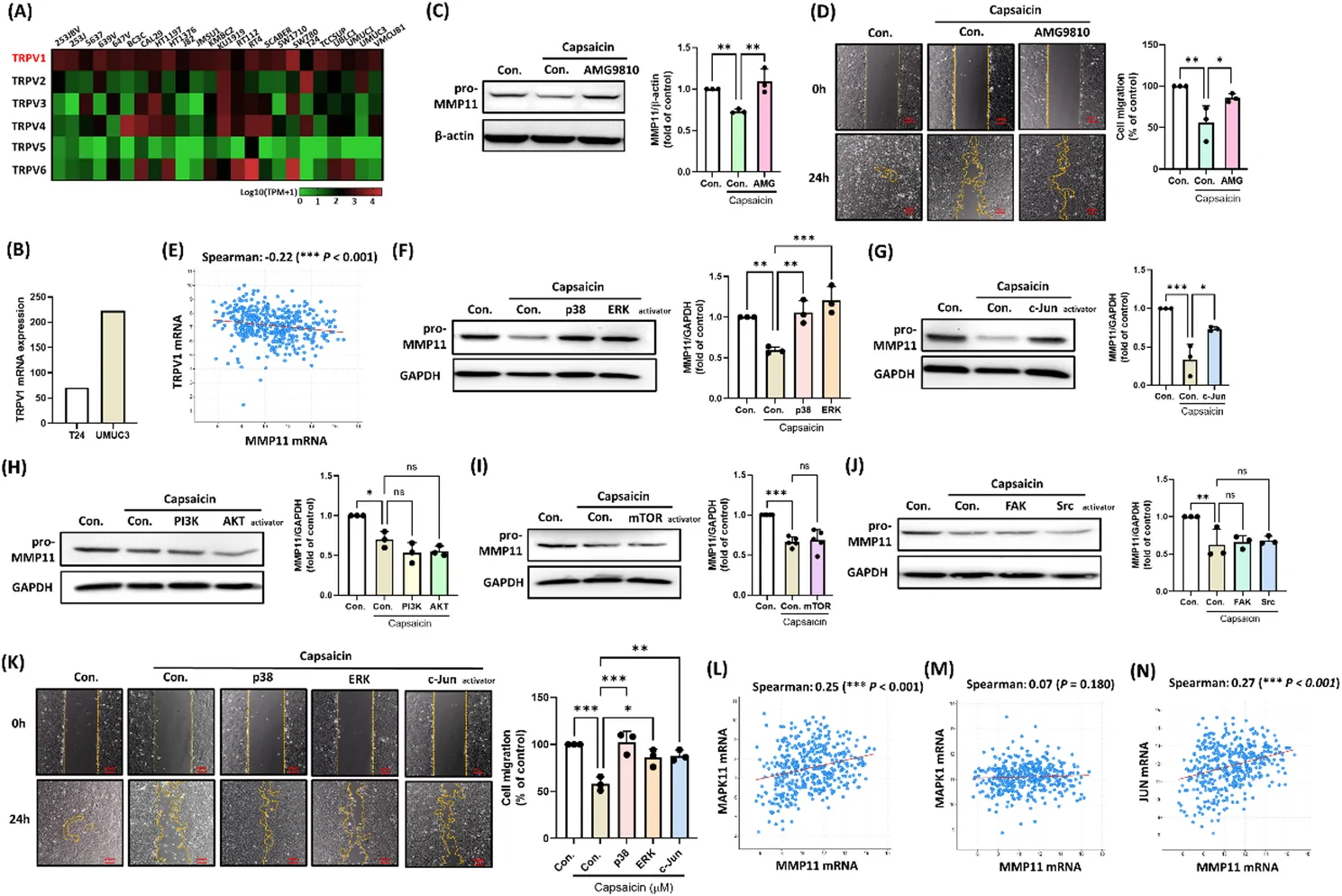
Capsaicin reduces the aggressiveness of BLCA cells through the involvement of the TRPV1 receptor and MAPK signaling pathways. (**A**) A heatmap representing the mRNA expression levels of TRPV1–TRPV6 in various BLCA cell lines, as analyzed using the CCLE database. (**B**) Expression levels of TRPV1 mRNA in BLCA cells were analyzed using the CCLE database. (**C**) Cells were pretreated with a TRPV1-specific inhibitor (AMG9810; 0.1 µg/mL) for 30 min, followed by capsaicin treatment for 24 h. The expression levels of MMP11 protein were analyzed through Western blot analysis. (**D**) A gap closure assay was conducted to assess cell motility upon cotreatment with capsaicin and AMG9810. Scale bar = 100 μm. (**E**) In human BLCA, MMP11 and TRPV1 were negatively correlated. (**F–J**) BLCA cells were pretreated with specific activators of p38 (anisomycin; 5 µM), ERK (ceramide C6; 10 µM), c-Jun (sphingosine-1-phosphate; 5 µM), PI3K (PI3-kinase activator; 5 µM), AKT (SC79; 1 µg/mL), mTOR (MHY1485; 10 µM), FAK (angiotensin II; 10 µM), or Src (YEE1; 10 µM) for 30 min, cells were then exposed to capsaicin for 24 h. MMP11 expression was assessed through Western blot analysis. (**K**) Gap closure assay was performed to analyze cell motility. Scale bar = 100 μm. (**L–N**) Positive correlations were observed between the expression levels of MMP11, MAPK11, JUN, and MAPK1 in BLCA tissues. NS, not significant; * *P* < 0.05, ** *P* < 0.01, and *** *P* < 0.001 compared with the control group, as determined by one-way ANOVA (**C, D, F-K**) or Spearman’s test (**E, L–N**)

Capsaicin is a highly specific agonist of the transient receptor potential vanilloid 1 (TRPV1) receptor, whose activation induces heat and pain sensations [46]; however, whether capsaicin modulates the aggressiveness of BLCA cells through TRPV1 signaling remains unclear. To address this issue, we first analyzed TRPV family expression using the CCLE database and found that, compared with other TRPV receptors, TRPV1 was markedly expressed in BLCA cell lines (Fig. 4A). The expression level of the TRPV1 receptor was upregulated in UMUC3 cells relative to that in T24 cells, indicating that UMUC3 cells exhibit a higher degree of sensitivity to capsaicin than T24 cells (Fig. 4B). Pretreatment of BLCA cells with the TRPV1 inhibitor (AMG9810) suppressed the capsaicin-mediated inhibition of MMP11 protein (Fig. 4C). Additionally, AMG9810 mitigated the capsaicin-mediated reduction of cell motility as well (Fig. 4D). BLCA tissue analysis revealed a negative correlation between the expression level of MMP11 and TRPV1 (Spearman coefficient: −0.22; Fig. 4E). Thus, capsaicin inhibits MMP11 expression through the TRPV1 receptor and thereby reduces the aggressiveness of BLCA cells.

A review of the literature revealed that the TRPV1 receptor induces signaling through several pathways, including the MAPK [47], PI3K/AKT/mTOR [48], and FAK/SRC pathways [49]. The administration of pathway-specific activators before capsaicin treatment revealed that MAPK signaling molecules, such as p38, extracellular signal-regulated kinase (ERK), and c-Jun, effectively interrupted the capsaicin-mediated reduction in MMP11 protein expression (Fig. 4F&G). The effects of other activators were not equally significant (Fig. 4H-J). The pre-treatment of BLCA cells with p38, ERK, and c-Jun activators counteracted the inhibitory effect of capsaicin on cell motility (Fig. 4K). Positive correlations were observed between MMP11, p38 (*MAPK11*) and c-Jun (*JUN*) in the BLCA tissues (Fig. 4L-N). These findings indicate that capsaicin regulates MMP11 expression and cell motility by modulating primarily the MAPK signaling molecules p38, ERK, and c-Jun.

**Fig. 5:**
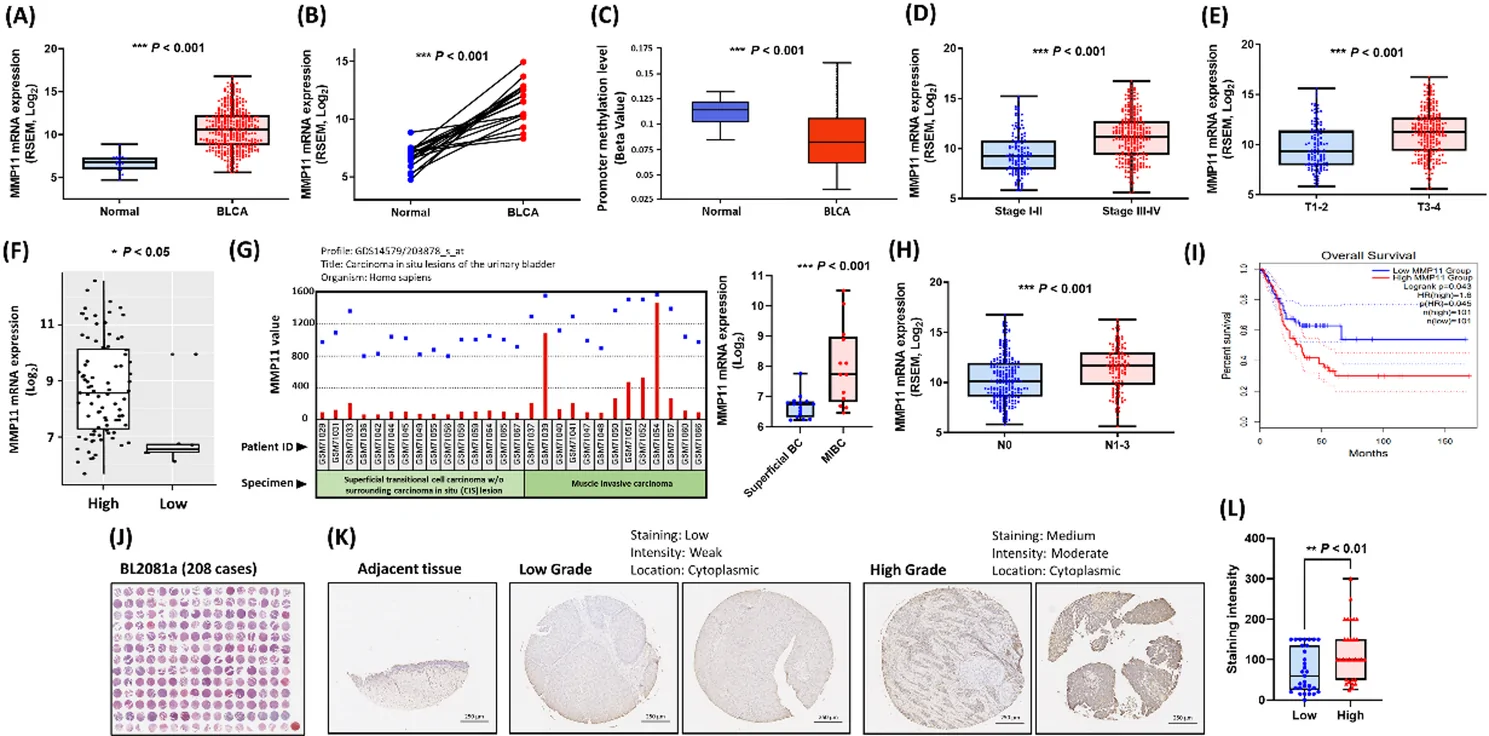
MMP11 represents a promising prognostic biomarker in BLCA. (**A&B**) MMP11 expression in normal and BLCA tissues was analyzed using data from TCGA-BLCA database. (**C**) Levels of MMP11 promoter methylation were analyzed using the UALCAN web server. (**D–F**) MMP11 expression in the clinical stage, T status, and high- or low-grade BLCA were analyzed using data from the TCGA-BLCA database. (**G**) BLCA profiles (GDS1479/203878_s_at) in GEO were used to analyze the expression level of MMP11 mRNA in carcinoma in situ tissues and MIBC tissues. (**H**) MMP11 expression in patients with or without BLCA lymphatic metastasis was analyzed using data from TCGA-BLCA database. (**I**) The rates of OS in patients with BLCA with high- or low-expression levels of MMP11 were analyzed using data from the GEPIA2 web server. (**J–L**) Immunohistochemical staining revealed MMP11 expression in a human BLCA tissue array (BL2081a). Scale bar = 250 μm. * *P* < 0.05, ** *P* < 0.01, and *** *P* < 0.001 compared with the control group, as determined by Student’s *t*-test (**A-H, L**) or Kaplan–Meier survival analysis (**I**)

### MMP11 is a promising prognostic marker of BLCA

To determine the potential of MMP11 as a prognostic marker of cancers, we investigated the likely significant associations between MMP11 expression levels and clinical characteristics specific to patients with BLCA. Analysis of the TCGA-BLCA database revealed that MMP11 mRNA expression levels were significantly higher in BLCA specimens (*n* = 408) than in normal tissue specimens (*n* = 19) (Fig. 5A). To minimize variations in gene expression across different individuals, we compared normal and cancer tissue samples obtained from the same group of patients. The expression level of MMP11 mRNA was significantly higher in all 19 cancer samples than in their normal tissue counterparts (Fig. 5B). Low levels of promoter methylation may advance gene transcription [50]. We observed that the methylation level in the MMP11 promoter was significantly reduced in cancer tissues (Fig. 5C). Moreover, MMP11 mRNA expression level was positively correlated with clinical stage and T status (Fig. 5D&E). High-grade BLCA tissues exhibited higher expression levels of MMP11 mRNA than did low-grade BLCA tissues (Fig. 5F).

Analysis of the NCBI GEO database revealed that MMP11 expression levels were significantly elevated in patients with muscle-invasive bladder cancer (MIBC) (Fig. 5G) and showed an increasing trend with advancing T status (Supplementary Fig. 2B). Moreover, high MMP11 expression was associated with lymph node metastasis (Fig. 5H). Patients with high MMP11 expression levels had significantly worse overall survival (OS) compared to those with low MMP11 expression levels (Fig. 5I), whereas no significant association was observed between MMP11 expression and disease-free survival (DFS) (Supplementary Fig. 2C). Finally, using a human BLCA tissue array (BL2081a) comprising 208 samples (Fig. 5J), we evaluated the protein level of MMP11 expression in BLCA tissues. MMP11 was primarily expressed in tumor tissues rather than adjacent tissues (Fig. 5K). Notably, high-grade tumor tissues exhibited higher expression levels of MMP11 than low-grade tumor tissues (Fig. 5L). These findings indicate that MMP11 expression is clinically significant and is positively correlated with clinical staging, MIBC, lymphatic metastasis, and poor prognosis in patients with BLCA. Therefore, MMP11 may be a prognostic marker of BLCA.

### Functional analyses for identifying the role of MMP11 in BLCA progression

**Fig. 6:**
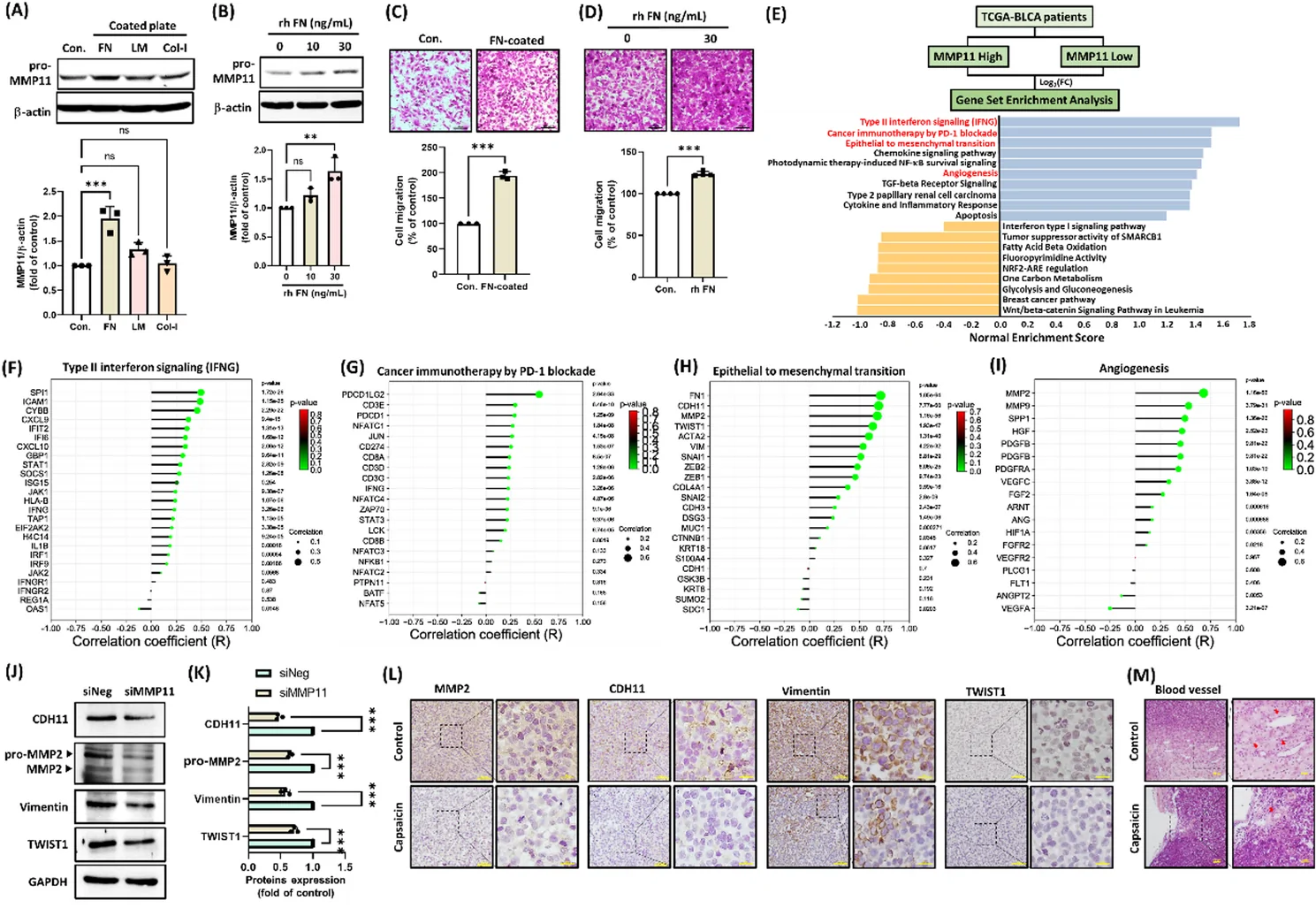
Functional analyses revealed the involvement of MMP11 in the progression of BLCA. (**A**) BLCA cells were seeded on ECM-coated plates for 24 h. Western blot analysis was used to measure MMP11 protein expression. (**B**) BLCA cells were treated with different concentrations of rh FN (0, 10, and 30 ng/mL) for 24 h, followed by the evaluation of MMP11 levels through Western blot analysis. (**C&D**) BLCA cells were seeded on FN-coated plates or incubated with rh FN for 24 h. Cell motility was assessed through Transwell migration assay. Scale bar = 100 μm. (**E**) Functional analysis of MMP11 was performed using The Cancer Genome Atlas database. (**F–I**) Correlation plots revealed both positive and negative correlations between MMP11 and those of genes associated with type II interferon signaling, cancer immunotherapy programmed cell death 1 blockade, epithelial–mesenchymal transition, and angiogenesis. (**J&K**) Expression of CDH11, MMP2, Vimentin, and Twist1 protein levels was detected in BLCA cells with or without MMP11 siRNA transfection. (**L**) Histologic sections of the tumors were stained with MMP2, CDH11, Vimentin and Twist1 in the mouse tumor tissues. Scale bar = 25, 100 μm. (**M**) H&E staining was used to observe blood vessels in tumor tissues. Scale bar = 50, 100 μm. NS, not significant; * *P* < 0.05, ** *P* < 0.01, and *** *P* < 0.001 compared with the control group, as determined by one-way ANOVA (**A, B**), Student’s *t*-test (**C, D**), Spearman’s test (**F-I**), or two-way ANOVA (**K**)

Next, we sought to elucidate how the tumor microenvironment contributes to elevated MMP11 expression levels in BLCA. Extracellular matrix proteins play crucial roles in inducing the expression of MMPs and affecting the aggressiveness of tumor cells [51–53]. BLCA cells were seeded on extracellular matrix (ECM) protein-coated plates, including fibronectin (FN), laminin (LM), and type I collagen (Col-I). The data revealed that fibronectin induced MMP11 protein expression (Fig. 6A). Treatment with different concentrations of human recombinant fibronectin (rh FN) also enhanced MMP11 expression (Fig. 6B). Both FN-coated plates and rh FN increased the motility of BLCA cells, indicating that FN can induce MMP11 expression in the tumor microenvironment, thereby enhancing the migratory ability of tumor cells (Fig. 6C&D).

Furthermore, human BLCA samples were stratified into high- and low-MMP11 expression groups. Functional analyses were subsequently performed to investigate the role of MMP11 in BLCA progression. The results revealed four functions to be strongly correlated with MMP11 expression levels: type II interferon signaling, cancer immunotherapy by PD-1 blockade, epithelial–mesenchymal transition (EMT) and angiogenesis (Fig. 6E). Furthermore, MMP11 expression was positively correlated with the expression of multiple genes associated with these four functions (Fig. 6F–I). Considering the significant effect of EMT on tumor progression and malignant metastasis [54], we conducted a Western blot analysis to investigate whether MMP11 regulates EMT function in BLCA cells. Treatment of cells with MMP11 siRNA resulted in the downregulation of EMT-related proteins, including CDH11, MMP2, Vimentin, and Twist1 (Fig. 6J&K). In capsaicin-treated tumor xenograft mouse model, the expression levels of MMP11-associated EMT and angiogenesis markers, including MMP2, CDH11, Vimentin, and Twist1, were decreased (Fig. 6L). We also observed that capsaicin reduced the infiltration of blood vessels into tumor tissue (Fig. 6M). Overall, our comprehensive analysis indicates MMP11 has a broad influence on EMT activity and angiogenesis in the tumor microenvironment of BLCA.

## Discussion

The antitumor effects of capsaicin on human cancer have been extensively researched [22, 30]. In the present study, we further differentiated between low and high concentrations of capsaicin to demonstrate its differential anticancer properties in BLCA. A high concentration of capsaicin directly induced apoptosis through the activation of caspase-3 and PARP, whereas a low concentration of capsaicin significantly reduced the MMP11-induced invasiveness of BLCA cells. Notably, a capsaicin engagement–specific TRPV1 receptor enhanced this mechanism by increasing the sensitivity of tumor cells to capsaicin. Capsaicin analogs with weaker side effects and lower toxicity (e.g., N-acyl-vanillylamide [N-AVAM]) were generated [55]. Preclinical studies have indicated that N-AVAM induces a higher level of apoptosis in melanoma, lung cancer, prostate cancer, cervical carcinoma, and breast cancer than does capsaicin in vitro [55–59]. The toxicity of N-AVAM was lower for normal cells than for cancer cells [55, 60]; N-AVAM leads to a greater reduction in side effects but an increased stability and growth-inhibitory propensity against cancer cells compared with capsaicin. Additional studies should conduct a comprehensive analysis of the potential benefits or limitations of capsaicin and its analogs in cancer therapy.

Many of the biological activities of capsaicin are mediated through activation of the TRPV1 channel, which is widely expressed in sensory neurons and vascular tissues [61, 62]. Activation of TRPV1 by capsaicin can stimulate the release of vasoactive neuropeptides such as calcitonin gene-related peptide (CGRP) and enhance endothelial nitric oxide production, leading to vasodilation and regulation of blood pressure [63]. Capsaicin has also been shown to inhibit platelet aggregation and blood coagulation [64]. In addition, dietary capsaicin consumption has been associated with beneficial effects on cardiovascular and metabolic regulation [65]. These observations indicate that capsaicin can modulate systemic physiological responses in humans. Further animal studies and clinical investigations will be necessary to determine whether therapeutically effective concentrations of capsaicin for anticancer therapy can be achieved without adversely affecting normal physiological functions.

TRPV1 is also gaining attention in the field of oncology research [66]. In the current study, we discovered that capsaicin inhibits the transmission of the MAPK signaling pathway through TRPV1, thereby negatively regulating the expression of MMP11 protein and mRNA. Consequently, the mobility and invasive ability of the BLCA cells were significantly reduced. In addition, we discovered that TRPV1 plays a crucial role in the sensitivity of BLCA cells to capsaicin. Another study similarly indicated that capsaicin enhances the antiproliferation efficacy of pirarubicin by activating TRPV1 and inhibiting the nuclear translocation of proliferating cell nuclear antigen in BLCA cells, with this being particularly notable in cells with high expression levels of TRPV1 [67]. Furthermore, TRPV1 is regarded as a tumor suppressor in clear cell renal cell carcinoma due to its close association with immune cell infiltration and positive correlation with several cancer-inhibiting genes, such as p53 and von Hippel-Lindau [68]. TRPV1 expression is negatively correlated with tumor proliferation, EMT, stemness, and oncogenic signatures across cancers [69]. Upon activation by its agonist, TRPV1 facilitates cancer cell death and suppresses cancer progression [70, 71]. Therefore, TRPV1 agonists may effectively induce apoptosis in cancer cells, which may have implications for the treatment of human cancers with high expression levels of TRPV1.

Several matrix metalloproteinase inhibitors have been developed by targeting MMPs in human cancers [72]. For example, a wide-spectrum nonpeptidic inhibitor called MMI 270 B has been developed that effectively inhibits MMP1, MMP2, and MMP3 [73, 74]. Furthermore, as an inhibitory agent, BAY12-9566 was reported to exhibit selectivity for specific MMPs, such as MMP2, MMP3, and MMP9 [75]. Another potential inhibitor known as Ro 32-3555 was reported to effectively suppress the activity of multitudinous MMPs, such as MMP1, MMP2, MMP3, MMP8, MMP9, and MMP13 [76]. Although clinical trials involving these compounds have not reported desired outcomes, the field of MMP inhibitor research has continually advanced. In the present study, we observed strong correlations between MMP11 expression and clinical staging as well as poor clinical outcomes. Through functional analyses, we identified various MMP11 expression–associated protumor functions, such as type II interferon signaling, EMT, and angiogenesis. Through in vitro analysis, we demonstrated that suppressing MMP11 expression significantly reduced the motility and invasiveness of BLCA cells and downregulated the expression of EMT-related proteins in these cells. Therefore, MMP11 appears to be a promising prognostic marker and therapeutic target for BLCA. We observed that capsaicin inhibited MMP11 expression both in vitro and in vivo, implying its potential for BLCA treatment. Additionally, capsaicin has demonstrated inhibitory effects on MMP2 and MMP9 expression in human papillary thyroid carcinoma [77]. Neovastat, a naturally derived MMP inhibitor sourced from shark cartilage, demonstrates noteworthy antitumor efficacy in breast cancer. This inhibitor exerts its function by suppressing MMP2, MMP9, and MMP12 [78]. Future studies should investigate whether natural MMP inhibitors offer improved MMP inhibition with fewer side effects in cancer treatment.

The MAPK signaling pathway plays a key role in regulating various aspects of tumor progression, including cell proliferation, motility, and invasion [79, 80]. Therefore, MAPK signaling molecules are attractive targets for cancer therapy. In the present study, the treatment of BLCA cells with the activators of p38, ERK, and c-Jun repressed the capsaicin-inhibited cell motility and MMP11 expression. In addition, treating BLCA cells with activators of p38, ERK, and c-Jun interrupted the inhibitory effects of capsaicin on cell motility and MMP11 expression. Moreover, the expression levels of p38, ERK, and c-Jun were positively correlated with that of MMP11 in human BLCA; this highlights the significance of MAPK signaling molecules as the upstream regulators of MMP11. Given the pivotal roles of MAPK signaling molecules in tumor progression, numerous clinical trials targeting these molecules are currently ongoing [81]. For example, ralimetinib (LY2228820) is an ATP-competitive inhibitor that selectively targets p38 MAPK α/β, and a phase 1b/2 clinical trial (NCT01663857) reported that when ralimetinib was comminated with gemcitabine and carboplatin, a modest enhancement was noted in progression-free survival among women with recurrent platinum-sensitive ovarian cancer [82]. Talmapimod (SCIO-469), an orally administered ATP-competitive inhibitor of p38α, is currently being tested in a phase 2 clinical trial (NCT00087867) as monotherapy or combination therapy (with Bortezomib) for relapsed multiple myeloma [83]. ASTX029, a synthetic small molecule inhibitor of ERK, inhibits the proliferation of cancer cells with *BRAF* or *RAS* mutations [84] and is currently being evaluated in a phase I/II clinical trial as an oral drug for advanced solid tumors (NCT03520075). Notably, although these MAPK inhibitors have exhibited potential in preclinical and clinical trials, further studies are required to evaluate their efficacy and safety for clinical applications.

### Conclusions

**Fig. 7:**
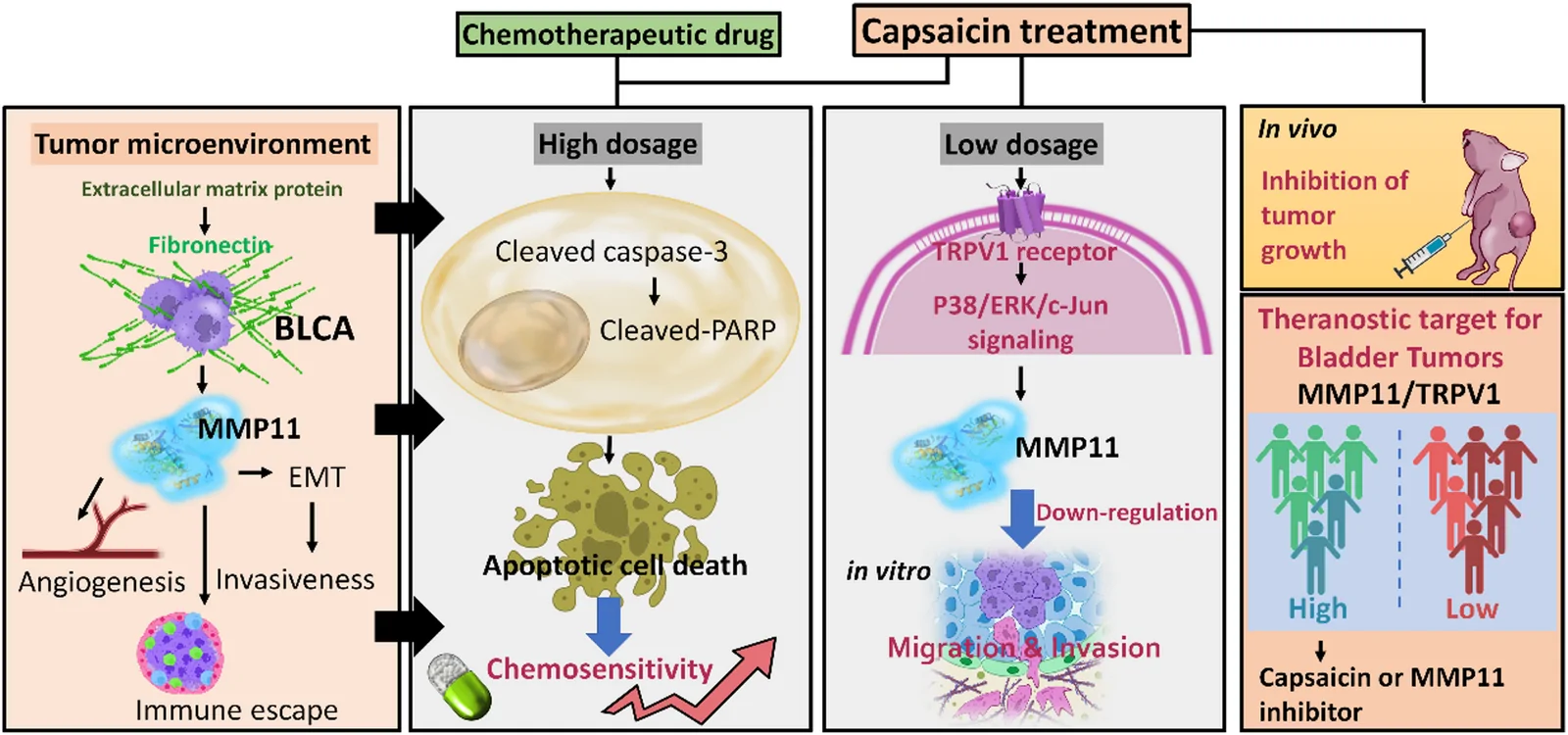
Clinical advantages of capsaicin in the treatment of human BLCA. ECM-FN in the BLCA tumor microenvironment upregulates MMP11, promoting cell invasiveness and angiogenesis. Under capsaicin treatment, apoptosis is induced via caspase-3 activation, and MMP11 is effectively targeted, resulting in reduced invasiveness and tumor progression. Capsaicin also suppresses tumor growth in vivo and enhances the efficacy of chemotherapeutic agents when used in combination. These findings suggest that MMP11 is a potential prognostic marker and therapeutic target, and that capsaicin may serve as a therapeutic option for BLCA patients with elevated MMP11 and TRPV1 expression

In human BLCA, within the tumor microenvironment, ECM-FN triggers the upregulation of MMP11 expression, subsequently promoting MMP11-mediated invasiveness and angiogenesis. Capsaicin appears to induce apoptosis in cancer cells through the activation of caspase-3, inhibit cancer cell invasion through the downregulation of MMP11 expression, and suppress the growth of tumors in vivo. When used in combination with traditional chemotherapeutic drugs, capsaicin enhances the drugs’ efficacy against BLCA. These findings suggest that MMP11 shows promise as both a prognostic marker and a potential therapeutic target in BLCA. Additionally, capsaicin holds potential as a therapeutic agent for patients with elevated MMP11 and TRPV1 expression levels (Fig. 7).

## Supplementary Information

Supplementary material 1.

## Data Availability

The datasets generated during and/or analyzed during the current study are available from the corresponding author on reasonable request.

## References

[1] Antoni S, et al. Bladder Cancer Incidence and Mortality: A Global Overview and Recent Trends. Eur Urol. 2017;71(1):96–108. 10.1016/j.eururo.2016.06.010 27370177

[2] Chamie K, et al. Recurrence of high-risk bladder cancer: a population-based analysis. Cancer. 2013;119(17):3219–27. 10.1002/cncr.28147 PMC3773281 23737352

[3] Aldousari S, Kassouf W. Update on the management of non-muscle invasive bladder cancer. Can Urol Assoc J. 2010;4(1):56–64. 10.5489/cuaj.777 PMC2812001 20165581

[4] van Ginkel N, et al. Survival outcomes of patients with muscle-invasive bladder cancer according to pathological response at radical cystectomy with or without neo-adjuvant chemotherapy: a case-control matching study. Int Urol Nephrol. 2022;54(12):3145–52. 10.1007/s11255-022-03339-6 PMC9606088 35997906

[5] Beigi A, et al. Survival Outcomes Associated with First and Second-Line Palliative Systemic Therapies in Patients with Metastatic Bladder Cancer. Curr Oncol. 2021;28(5):3812–24. 10.3390/curroncol28050325 PMC8534510 34677243

[6] Cabral-Pacheco GA et al. *The Roles of Matrix Metalloproteinases and Their Inhibitors in Human Diseases.* Int J Mol Sci, 2020. 21(24):9739. 10.3390/ijms21249739 PMC7767220 33419373

[7] Quintero-Fabian S, et al. Role of Matrix Metalloproteinases in Angiogenesis and Cancer. Front Oncol. 2019;9:1370. 10.3389/fonc.2019.01370 PMC6915110 31921634

[8] Page-McCaw A, Ewald AJ, Werb Z. Matrix metalloproteinases and the regulation of tissue remodelling. Nat Rev Mol Cell Biol. 2007;8(3):221–33. 10.1038/nrm2125 PMC2760082 17318226

[9] Caley MP, Martins VL, O’Toole EA. Metalloproteinases and Wound Healing. Adv Wound Care (New Rochelle). 2015;4(4):225–34. 10.1089/wound.2014.0581 PMC4397992 25945285

[10] Gonzalez-Avila G, et al. Matrix metalloproteinases participation in the metastatic process and their diagnostic and therapeutic applications in cancer. Crit Rev Oncol Hematol. 2019;137:57–83. 10.1016/j.critrevonc.2019.02.010 31014516

[11] Wang SW, et al. Melatonin impedes prostate cancer metastasis by suppressing MMP-13 expression. J Cell Physiol. 2021;236(5):3979–90. 10.1002/jcp.30150 33251599

[12] Chen WC, et al. Bone sialoprotein promotes lung cancer osteolytic bone metastasis via MMP14-dependent mechanisms. Biochem Pharmacol. 2023;211:115540. 10.1016/j.bcp.2023.115540 37028462

[13] Kim HS, et al. High MMP-11 expression associated with low CD8 + T cells decreases the survival rate in patients with breast cancer. PLoS ONE. 2021;16(5):e0252052. 10.1371/journal.pone.0252052 PMC8153507 34038440

[14] Yang H, et al. Matrix Metalloproteinase 11 Is a Potential Therapeutic Target in Lung Adenocarcinoma. Mol Ther Oncolytics. 2019;14:82–93. 10.1016/j.omto.2019.03.012 PMC6477516 31024988

[15] Pang L, et al. Elevated serum levels of MMP-11 correlate with poor prognosis in colon cancer patients. Cancer Biomark. 2016;16(4):599–607. 10.3233/CBM-160601 PMC13016514 27002762

[16] Deng H, et al. Matrix metalloproteinase 11 depletion inhibits cell proliferation in gastric cancer cells. Biochem Biophys Res Commun. 2005;326(2):274–81. 10.1016/j.bbrc.2004.11.027 15582574

[17] Jia L, et al. siRNA targeted against matrix metalloproteinase 11 inhibits the metastatic capability of murine hepatocarcinoma cell Hca-F to lymph nodes. Int J Biochem Cell Biol. 2007;39(11):2049–62. 10.1016/j.biocel.2007.05.023 17627864

[18] Basset P, et al. Matrix metalloproteinases as stromal effectors of human carcinoma progression: therapeutic implications. Matrix Biol. 1997;15(8–9):535–41. 10.1016/s0945-053x(97)90028-7 9138286

[19] Jones LE, et al. Comprehensive analysis of matrix metalloproteinase and tissue inhibitor expression in pancreatic cancer: increased expression of matrix metalloproteinase-7 predicts poor survival. Clin Cancer Res. 2004;10(8):2832–45. 10.1158/1078-0432.ccr-1157-03 15102692

[20] Thewes M, et al. Stromelysin-3 (ST-3) mRNA expression in colorectal carcinomas. Localization and clinicopathologic correlations. Diagn Mol Pathol. 1996;5(4):284–90. 10.1097/00019606-199612000-00010 8955621

[21] Hsieh CY et al. Impact of Matrix Metalloproteinase-11 Gene Polymorphisms on Biochemical Recurrence and Clinicopathological Characteristics of Prostate Cancer. Int J Environ Res Public Health, 2020. 17(22):8603. 10.3390/ijerph17228603 PMC7699319 33228130

[22] Chapa-Oliver AM, Mejia-Teniente L. Capsaicin: From Plants to a Cancer-Suppressing Agent. Molecules, 2016. 21(8):931. 10.3390/molecules21080931 PMC6274000 27472308

[23] Wallace M, et al. A Randomized, Double-Blind, Placebo-Controlled, Crossover Study of the T-Type Calcium Channel Blocker ABT-639 in an Intradermal Capsaicin Experimental Pain Model in Healthy Adults. Pain Med. 2016;17(3):551–60. 10.1093/pm/pnv068 26814294

[24] Wang H, et al. Effect of morphine and pregabalin compared with diphenhydramine hydrochloride and placebo on hyperalgesia and allodynia induced by intradermal capsaicin in healthy male subjects. J Pain. 2008;9(12):1088–95. 10.1016/j.jpain.2008.05.013 19038771

[25] Rosa A, et al. Antioxidant activity of capsinoids. J Agric Food Chem. 2002;50(25):7396–401. 10.1021/jf020431w 12452665

[26] Kim CS, et al. Capsaicin exhibits anti-inflammatory property by inhibiting IkB-a degradation in LPS-stimulated peritoneal macrophages. Cell Signal. 2003;15(3):299–306. 10.1016/s0898-6568(02)00086-4 12531428

[27] Zheng J et al. Dietary capsaicin and its anti-obesity potency: from mechanism to clinical implications. Biosci Rep, 2017. 37(3):BSR20170286. 10.1042/BSR20170286 PMC5426284 28424369

[28] Caterina MJ, et al. The capsaicin receptor: a heat-activated ion channel in the pain pathway. Nature. 1997;389(6653):816–24. 10.1038/39807 9349813

[29] Brito R, et al. TRPV1: A Potential Drug Target for Treating Various Diseases. Cells. 2014;3(2):517–45. 10.3390/cells3020517 PMC4092862 24861977

[30] Clark R, Lee SH. Anticancer Properties of Capsaicin Against Human Cancer. Anticancer Res. 2016;36(3):837–43. 26976969

[31] Oyagbemi AA, Saba AB, Azeez OI. Capsaicin: a novel chemopreventive molecule and its underlying molecular mechanisms of action. Indian J Cancer. 2010;47(1):53–8. 10.4103/0019-509X.58860 20071791

[32] Hong ZF, et al. Capsaicin Enhances the Drug Sensitivity of Cholangiocarcinoma through the Inhibition of Chemotherapeutic-Induced Autophagy. PLoS ONE. 2015;10(5):e0121538. 10.1371/journal.pone.0121538 PMC4416771 25933112

[33] Berger A, et al. Oral capsaicin provides temporary relief for oral mucositis pain secondary to chemotherapy/radiation therapy. J Pain Symptom Manage. 1995;10(3):243–8. 10.1016/0885-3924(94)00130-D 7629418

[34] Ellison N, et al. Phase III placebo-controlled trial of capsaicin cream in the management of surgical neuropathic pain in cancer patients. J Clin Oncol. 1997;15(8):2974–80. 10.1200/JCO.1997.15.8.2974 9256142

[35] Ho CY, et al. Miconazole induces protective autophagy in bladder cancer cells. Environ Toxicol. 2021;36(2):185–93. 10.1002/tox.23024 32981224

[36] Chou KY, et al. Thrombospondin-4 promotes bladder cancer cell migration and invasion via MMP2 production. J Cell Mol Med. 2021;25(13):6046–55. 10.1111/jcmm.16463 PMC8406484 34142438

[37] Hwang TI, et al. Hsa-miR-30a-3p overcomes the acquired protective autophagy of bladder cancer in chemotherapy and suppresses tumor growth and muscle invasion. Cell Death Dis. 2022;13(4):390. 10.1038/s41419-022-04791-z PMC9023440 35449123

[38] Bort A, et al. Capsaicin exerts synergistic antitumor effect with sorafenib in hepatocellular carcinoma cells through AMPK activation. Oncotarget. 2017;8(50):87684–98. 10.18632/oncotarget.21196 PMC5675664 29152112

[39] Steele TM, et al. Cisplatin-induced increase in heregulin 1 and its attenuation by the monoclonal ErbB3 antibody seribantumab in bladder cancer. Sci Rep. 2023;13(1):9617. 10.1038/s41598-023-36774-1 PMC10267166 37316561

[40] Porter AG, Janicke RU. Emerging roles of caspase-3 in apoptosis. Cell Death Differ. 1999;6(2):99–104. 10.1038/sj.cdd.4400476 10200555

[41] Zhang F, Lau SS, Monks TJ. A dual role for poly(ADP-ribose) polymerase-1 during caspase-dependent apoptosis. Toxicol Sci. 2012;128(1):103–14. 10.1093/toxsci/kfs142 PMC3491962 22523229

[42] Nurgali K, Jagoe RT, Abalo R. Editorial: Adverse Effects of Cancer Chemotherapy: Anything New to Improve Tolerance and Reduce Sequelae? Front Pharmacol. 2018;9:245. 10.3389/fphar.2018.00245 PMC5874321 29623040

[43] Entschladen F, et al. Tumour-cell migration, invasion, and metastasis: navigation by neurotransmitters. Lancet Oncol. 2004;5(4):254–8. 10.1016/S1470-2045(04)01431-7 15050959

[44] Min KW, et al. Diagnostic and prognostic relevance of MMP-11 expression in the stromal fibroblast-like cells adjacent to invasive ductal carcinoma of the breast. Ann Surg Oncol. 2013;20(Suppl 3):S433–42. 10.1245/s10434-012-2734-3 23115007

[45] Pan C, et al. Matrix Metalloproteinase 11 Promotes Migration and Invasion of Colorectal Cancer by Elevating Slug Protein. Int J Med Sci. 2024;21(11):2170–88. 10.7150/ijms.98007 PMC11373555 39239548

[46] Tominaga M, Julius D. Capsaicin receptor in the pain pathway. Jpn J Pharmacol. 2000;83(1):20–4. 10.1254/jjp.83.20 10887936

[47] Chen M et al. Mitogen-Activated Protein Kinase and Intracellular Polyamine Signaling Is Involved in TRPV1 Activation-Induced Cardiac Hypertrophy. J Am Heart Assoc, 2016. 5(8):e003718. 10.1161/JAHA.116.003718 PMC5015292 27473037

[48] Ching LC, et al. Molecular mechanisms of activation of endothelial nitric oxide synthase mediated by transient receptor potential vanilloid type 1. Cardiovasc Res. 2011;91(3):492–501. 10.1093/cvr/cvr104 21493704

[49] Liu Y, et al. TRPV1 Antagonist Prevents Neonatal Sevoflurane-Induced Synaptic Abnormality and Cognitive Impairment in Mice Through Regulating the Src/Cofilin Signaling Pathway. Front Cell Dev Biol. 2021;9:684516. 10.3389/fcell.2021.684516 PMC8293754 34307363

[50] Moore LD, Le T, Fan G. DNA methylation its basic function Neuropsychopharmacol. 2013;38(1):23–38. 10.1038/npp.2012.112 PMC3521964 22781841

[51] Pal S, et al. Extracellular matrix protein laminin induces matrix metalloproteinase-9 in human breast cancer cell line mcf-7. Cancer Microenviron. 2014;7(1–2):71–8. 10.1007/s12307-014-0146-6 PMC4150877 24858419

[52] Sen T, et al. Fibronectin induces matrix metalloproteinase-9 (MMP-9) in human laryngeal carcinoma cells by involving multiple signaling pathways. Biochimie. 2010;92(10):1422–34. 10.1016/j.biochi.2010.07.005 20638438

[53] Borrirukwanit K, et al. The type I collagen induction of MT1-MMP-mediated MMP-2 activation is repressed by alphaVbeta3 integrin in human breast cancer cells. Matrix Biol. 2007;26(4):291–305. 10.1016/j.matbio.2006.10.014 17175151

[54] Heerboth S, et al. EMT and tumor metastasis. Clin Transl Med. 2015;4:6. 10.1186/s40169-015-0048-3 PMC4385028 25852822

[55] Richbart SD, et al. Nonpungent N-AVAM Capsaicin Analogues and Cancer Therapy. J Med Chem. 2021;64(3):1346–61. 10.1021/acs.jmedchem.0c01679 PMC10442063 33508189

[56] Tuoya, et al. Apoptosis induction by dohevanil, a DHA substitutive analog of capsaicin, in MCF-7 cells. Life Sci. 2006;78(13):1515–9. 10.1016/j.lfs.2005.07.019 16260002

[57] Pereira GJV, et al. Capsaicin-like analogue induced selective apoptosis in A2058 melanoma cells: Design, synthesis and molecular modeling. Bioorg Med Chem. 2019;27(13):2893–904. 10.1016/j.bmc.2019.05.020 31104785

[58] Friedman JR, et al. Anticancer Activity of Natural and Synthetic Capsaicin Analogs. J Pharmacol Exp Ther. 2018;364(3):462–73. 10.1124/jpet.117.243691 PMC5803642 29246887

[59] Brown KC, et al. Anticancer Activity of Region B Capsaicin Analogs. J Med Chem. 2023;66(7):4294–323. 10.1021/acs.jmedchem.2c01594 PMC10108357 37000154

[60] Macho A, et al. Non-pungent capsaicinoids from sweet pepper synthesis and evaluation of the chemopreventive and anticancer potential. Eur J Nutr. 2003;42(1):2–9. 10.1007/s00394-003-0394-6 12594536

[61] Maximiano TKE, et al. TRPV1: Receptor structure, activation, modulation and role in neuro-immune interactions and pain. Cell Calcium. 2024;119:102870. 10.1016/j.ceca.2024.102870 38531262

[62] Negri S et al. Endothelial TRPV1 as an Emerging Molecular Target to Promote Therapeutic Angiogenesis. Cells, 2020. 9(6):1341. 10.3390/cells9061341 PMC7349285 32471282

[63] Fattori V et al. Capsaicin: Current Understanding of Its Mechanisms and Therapy of Pain and Other Pre-Clinical and Clinical Uses. Molecules, 2016. 21(7):844. 10.3390/molecules21070844 PMC6273101 27367653

[64] Adams MJ, Ahuja KD, Geraghty DP. Effect of capsaicin and dihydrocapsaicin on in vitro blood coagulation and platelet aggregation. Thromb Res. 2009;124(6):721–3. 10.1016/j.thromres.2009.05.001 19476975

[65] Sun F, Xiong S, Zhu Z. Dietary Capsaicin Protects Cardiometabolic Organs Dysfunct Nutrients, 2016. 8(5):174. 10.3390/nu8050174 PMC4882656 27120617

[66] Bujak JK, et al. Inflammation, Cancer and Immunity-Implication of TRPV1 Channel. Front Oncol. 2019;9:1087. 10.3389/fonc.2019.01087 PMC6805766 31681615

[67] Zheng L, et al. Capsaicin enhances anti-proliferation efficacy of pirarubicin via activating TRPV1 and inhibiting PCNA nuclear translocation in 5637 cells. Mol Med Rep. 2016;13(1):881–7. 10.3892/mmr.2015.4623 26648574

[68] Zheng L, et al. TRPV1 acts as a Tumor Suppressor and is associated with Immune Cell Infiltration in Clear Cell Renal Cell Carcinoma: evidence from integrated analysis. J Cancer. 2020;11(19):5678–88. 10.7150/jca.45918 PMC7477432 32913462

[69] Nie R, Liu Q, Wang X. *TRPV1 Is a Potential Tumor Suppressor for Its Negative Association with Tumor Proliferation and Positive Association with Antitumor Immune Responses in Pan-Cancer.* J Oncol, 2022. 2022: p. 6964550. 10.1155/2022/6964550 PMC9596243 36304985

[70] Vercelli C, et al. Transient receptor potential vanilloid 1 expression and functionality in mcf-7 cells: a preliminary investigation. J Breast Cancer. 2014;17(4):332–8. 10.4048/jbc.2014.17.4.332 PMC4278051 25548580

[71] Wu TT, et al. Consequences of activating the calcium-permeable ion channel TRPV1 in breast cancer cells with regulated TRPV1 expression. Cell Calcium. 2014;56(2):59–67. 10.1016/j.ceca.2014.04.006 24889371

[72] Winer A, Adams S, Mignatti P. Matrix Metalloproteinase Inhibitors in Cancer Therapy: Turning Past Failures Into Future Successes. Mol Cancer Ther. 2018;17(6):1147–55. 10.1158/1535-7163.MCT-17-0646 PMC5984693 29735645

[73] Chaudhary AK, et al. Genetic polymorphisms of matrix metalloproteinases and their inhibitors in potentially malignant and malignant lesions of the head and neck. J Biomed Sci. 2010;17(1):10. 10.1186/1423-0127-17-10 PMC2846899 20152059

[74] Levitt NC, et al. Phase I and pharmacological study of the oral matrix metalloproteinase inhibitor, MMI270 (CGS27023A), in patients with advanced solid cancer. Clin Cancer Res. 2001;7(7):1912–22. 11448904

[75] Erlichman C, et al. Phase I study of the matrix metalloproteinase inhibitor, BAY 12-9566. Ann Oncol. 2001;12(3):389–95. 10.1023/a:1011183905848 11332153

[76] Close DR. Matrix metalloproteinase inhibitors in rheumatic diseases. Ann Rheum Dis. 2001;60(Suppl 3):iii62–7. 10.1136/ard.60.90003.iii62 PMC1766680 11890658

[77] Xu S, et al. Capsaicin inhibits the metastasis of human papillary thyroid carcinoma BCPAP cells through the modulation of the TRPV1 channel. Food Funct. 2018;9(1):344–54. 10.1039/c7fo01295k 29185571

[78] Falardeau P, et al. Neovastat, a naturally occurring multifunctional antiangiogenic drug, in phase III clinical trials. Semin Oncol. 2001;28(6):620–5. 10.1016/s0093-7754(01)90035-1 11740820

[79] Guo YJ, et al. ERK/MAPK signalling pathway and tumorigenesis. Exp Ther Med. 2020;19(3):1997–2007. 10.3892/etm.2020.8454 PMC7027163 32104259

[80] Yang M, Huang CZ. Mitogen-activated protein kinase signaling pathway and invasion and metastasis of gastric cancer. World J Gastroenterol. 2015;21(41):11673–9. 10.3748/wjg.v21.i41.11673 PMC4631968 26556994

[81] Braicu C et al. A Comprehensive Review on MAPK: A Promising Therapeutic Target in Cancer. Cancers (Basel), 2019. 11(10):1618. 10.3390/cancers11101618 PMC6827047 31652660

[82] Vergote I, et al. A randomized, double-blind, placebo-controlled phase 1b/2 study of ralimetinib, a p38 MAPK inhibitor, plus gemcitabine and carboplatin versus gemcitabine and carboplatin for women with recurrent platinum-sensitive ovarian cancer. Gynecol Oncol. 2020;156(1):23–31. 10.1016/j.ygyno.2019.11.006 31791552

[83] Phan T, et al. P38 kinase in gastrointestinal cancers. Cancer Gene Ther; 2023. 10.1038/s41417-023-00622-1 PMC10501902 37248432

[84] Munck JM, et al. ASTX029, a Novel Dual-mechanism ERK Inhibitor, Modulates Both the Phosphorylation and Catalytic Activity of ERK. Mol Cancer Ther. 2021;20(10):1757–68. 10.1158/1535-7163.MCT-20-0909 34330842

