# Supplementary material for: Capsaicin enhances chemosensitivity and mitigates MMP11-driven aggressiveness in human bladder cancer
Source: Cancer Cell Int. 2026 Jul 24;26:346. doi: 10.1186/s12935-026-04397-x (PMC13685141; doi:10.1186/s12935-026-04397-x)
Supplement: Supplementary file 1 [file 12935_2026_4397_MOESM1_ESM.docx]

**Supplementary Figure 1. Capsaicin inhibits BCL2 protein expression in human BLCA cells.** (A&B) UMUC3 cells were treated with different concentrations of capsaicin for 24 h. BCL2 and BAX expression was analyzed by Western blot. (C) Bax/Bcl-2 ratio was calculated. **P* < 0.05 and ****P* < 0.001 compared with the control group.

**Supplementary Figure 2. Clinical significance of MMP11 expression in human BLCA.** (A) Heatmap showing the expression profiles of the top 10 ranked genes in BLCA tissues, generated from the TCGA-BLCA dataset and analyzed using the UALCAN web portal. (B) BLCA expression profiles from the GEO dataset (GDS4456/203878_s_at) were used to analyze MMP11 mRNA expression across different T statuses in BLCA patients. (C) Disease-free survival (DFS) rates of BLCA patients with high or low MMP11 expression were analyzed using data from the GEPIA2 web server. ***P* < 0.01 compared with the control group.
